# The Impact of the COVID-19 Pandemic on the Experiences and Operations of Sponsors of the Summer Food Service Program in Maryland, USA: A Multiphase Mixed Methods Study

**DOI:** 10.3390/nu15071628

**Published:** 2023-03-27

**Authors:** Stacy V. Lu, Kaitlyn M. Harper, Yoyo Ding, Jordan Everett, Julia Gross, Rachael Borman, Karen Medina-Perez, Brielle Pinzini, Michael J. Wilson, Susan M. Gross

**Affiliations:** 1 Department of Population, Family and Reproductive Health, Johns Hopkins Bloomberg School of Public Health, 615 N Wolfe St., Baltimore, MD 21205, USA; 2 Department of International Health Human Nutrition Program, Johns Hopkins Bloomberg School of Public Health, 615 N Wolfe St., Baltimore, MD 21205, USA; 3 Maryland Hunger Solutions, 711 W 40th St., Suite 360, Baltimore, MD 21211, USA

**Keywords:** COVID-19, pandemic, child nutrition, out-of-school time nutrition, summer meals, food insecurity, federal nutrition programs, summer food service program

## Abstract

During the COVID-19 pandemic, the Summer Food Service Program (SFSP) was allowed to operate in untraditional non-summer months to ensure children did not lose access to free and reduced-priced nutritious meals when schools were mandated to close in the United States. This study assessed the impact of the pandemic on the operations and experiences of Summer Food Service Program (SFSP) sponsors in the state of Maryland during the COVID-19 pandemic in 2020 (Phase I) and 2021 (Phase II). This study used a multiphase explanatory sequential mixed methods design with qualitative prioritization. Maryland SFSP sponsors completed an online survey (Phase I: n = 27, Phase II: n = 30), and semi-structured in-depth interviews were conducted with a subset of sponsors who completed the survey (Phase I: n = 12, Phase II: n = 7). Inductive and deductive analyses were used for qualitative data, and descriptive statistics were used for quantitative data. The COVID-19 pandemic caused SFSP sponsors to change their operations. Sponsors were primarily concerned about staff safety/burnout and decreased participation. Sponsors perceived waivers implemented by the United States Department of Agriculture to be crucial in enabling them to serve meals to children during the pandemic. The findings from our study support advocacy efforts to permanently implement waivers and provide free school meals for all children.

## 1. Introduction

Before the pandemic, school-aged children from low-income households could access free- and reduced-price meals at school through the National School Lunch Program (NSLP) and School Breakfast Program (SBP) in the United States (US). During out-of-school times, such as the summer months (i.e., June, July, August) and mandated school closures (e.g., due to inclement weather, natural disaster, or pandemic), children lose access to these healthy meals [1]. The Summer Food Service Program (SFSP) is a federal nutrition program that aims to address this meal gap during out-of-school times. The SFSP is overseen by state agencies and implemented by local sponsors—including public and private school districts, government agencies, and faith-based and non-profit organizations—who distribute meals to children at one or more sites. 

In March 2020, several states mandated school closures due to COVID-19-related safety concerns. As a result, over 900,000 children in the state of Maryland lost access to school meals, including approximately 384,000 children who were eligible for free and reduced-priced meals [2], and the proportion of households with children experiencing food insecurity increased from approximately 15% to 43% in the state of Maryland [3,4]. The United States Department of Agriculture (USDA) implemented nationwide waivers that allowed sponsors to serve meals to children year-round through the SFSP as well as several other program-specific waivers to accommodate pandemic-related changes, such as allowing sponsors to serve to-go meals (called non-congregate meals) to adhere to physical distancing guidelines [5]. In Maryland, the total number of meals served during the summer months increased from approximately three million in 2019 to over nine million in 2020 [6]. SFSP sponsors were tasked with making unprecedented changes in their operations to accommodate the stark increase in the number of meals served while addressing pandemic-related challenges. 

### The Current Study

This study used mixed methods to examine the impact of the COVID-19 pandemic on the operations and experiences of Maryland SFSP sponsors in 2020 and 2021. The integration of quantitative and qualitative methods in mixed methods research allows for deeper investigation, provides more context, and allows for a complete understanding of data [7]. Quantitative research strands were used to describe barriers (challenges and concerns) and facilitators to operating the SFSP during the pandemic, while qualitative research strands were used to elaborate and explain the results of the quantitative strands. This study aimed to address the following research questions:

1. What were the sponsors’ barriers and facilitators to operating the SFSP during the pandemic?
2. How and why did those barriers and facilitators exist?

## 2. Materials and Methods

### 2.1. Study Design

**Figure 1:**
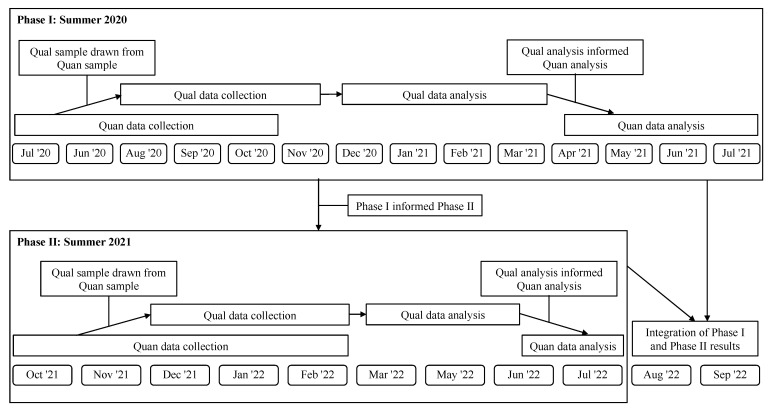
Visual display of multiphase explanatory sequential mixed methods study design.

This study used a multiphase explanatory sequential mixed methods design (quan → qual) (Figure 1). Phases I and II focused on sponsors’ opinions and experiences in 2020 and 2021, respectively. The data collection and analytical procedures in Phase II were built on and informed by Phase I. In both phases, quantitative survey data were collected and explained by qualitative data from semi-structured in-depth interviews (IDI). At the time of the study, little was known about the pandemic’s impacts on federal nutrition programs. Qualitative data were prioritized in this study to allow exploration and identification of key constructs important to sponsors during the pandemic grounded in their views and experiences. Quantitative data from the survey were selected for analysis based on themes that emerged from the qualitative analysis [7,8]. In explanatory sequential mixed methods designs, quantitative and qualitative constructs should align [7,8]. Data from both the survey (quan) and IDI (qual) addressed the following constructs regarding the impact of the COVID-19 pandemic on summer meals service in Phase I and Phase II:

- What challenges and concerns did sponsors experience during the pandemic?
- Why did sponsors face those challenges and concerns?
- What helped sponsors to address those challenges and concerns (facilitators)?

Both phases of this project were considered exempt (non-human subjects research) by the university’s Institutional Review Board.

### 2.2. Research Team

The research team included researchers at the Johns Hopkins Bloomberg School of Public Health and members of Maryland Hunger Solutions, a local anti-hunger advocacy organization. Three members (SVL, KMH, SG) of the research team have extensive experience and training in human nutrition and nutrition policy research. Over the two-year span of the study, four members (JE, RB, KMP, BP) were research interns or Americorps fellows with MDHS who assisted with at least one phase. Two members (JG, MJW) of the team have extensive experience with anti-hunger advocacy and work closely with the Maryland State Department of Education, child nutrition programs, and community leaders. Our partners at MDHS have pre-existing relationships with SFSP sponsors, which we leveraged to assist with survey and interview recruitment. Several members of the team (SVL, KMH, JG, MJW, SG) previously collaborated on an evaluation of the impact of the rescission of waivers on summer meals [6]. SVL conducted interviews in both phases; KMH and YD conducted interviews only in Phase I or Phase II, respectively. Three members of the team (SVL, KMH, YD) were trained and experienced in qualitative and quantitative research methods, and two members (SVL, KMH) had specific training and experience with mixed methods research. The first author led the mixing of qualitative and quantitative analyses.

### 2.3. Sampling and Data Collection

Data for the quantitative strands were collected first in both phases using total population sampling. Contact information for each sponsor was obtained from publicly available information (e.g., the school website). In each phase, we administered a one-time online survey using Qualtrics XM Survey Software, Versions July 2020 [9] and October 2021 [10], to SFSP sponsors who served meals in the summer (June, July, and August). Survey questions were adapted from the Nutrition and Obesity Policy Research and Evaluation Network COVID-19 Food and Nutrition Work Group survey repository [11], focusing on sponsors’ perceived barriers and facilitators to serving summer meals during the pandemic. The Phase II survey included additional questions about sponsors’ experiences with the transition to in-person meal service. The survey was open from July 2020 to October 2020 and October 2021 to February 2022 in Phase I and II, respectively. The study team followed up with sponsors three times via email and phone to complete the survey. 

In both phases, data for the qualitative strands were collected after quantitative data collection. A convenience sample of IDI participants was obtained from the survey sample, a common mixing method in a sequential explanatory design [8,12]. Interviews were conducted with a subset of SFSP sponsors who indicated on the survey that they would be willing to participate. The interview guides included questions addressing sponsors’ experiences operating their summer meals programs during the pandemic, challenges or barriers, facilitators to success, and sponsors’ opinions about the pandemic-related waivers and flexibilities, in addition to future policy recommendations. The interview guide used in Phase II included additional questions to explore sponsors’ experiences with the transition to in-person service. Interviews were conducted from August 2020 to November 2020 in Phase I and December 2021 to February 2022 in Phase II. Interviews were transcribed verbatim by a professional transcription service and verified against the audio by researchers.

### 2.4. Analysis

The following section describes the analytical procedures for the qualitative and quantitative data and how they were mixed. 

#### 2.4.1. Qualitative Analysis

In both phases, qualitative data were analyzed first by two researchers to guide and inform the interpretation of the quantitative data. MAXQDA Analytics Pro 2020 [13] and MAXQDA Analytics Pro 2022 [14] were used to assist with the organization and management of qualitative data. After each interview, interviewers wrote reflexive memos to document thoughts, lingering questions, and emerging patterns [15,16].

In Phase I, inductive and deductive approaches were used to analyze the interviews [15,16,17]. Two researchers first coded all twelve transcripts section-by-section and regularly met to develop the initial codebook. All transcripts were used in this phase to serve as an opportunity for both researchers to gain familiarity with the data through close reading; this was especially important for interviews where one researcher was not present and, therefore, unfamiliar with the data. Next, each researcher single-coded six transcripts and met throughout the process to compare coding and finalize the codebook. 

In Phase II, deductive approaches were used to analyze the interviews and specifically elaborate on areas where the quantitative and qualitative data diverged in Phase I. The final codebook from Phase I was used as the initial codebook in Phase II. The researchers followed a similar analysis process as was used in Phase I.

#### 2.4.2. Quantitative Analysis

Analysis of survey items focused on qualitative themes which converged or diverged from each other. All statistical analyses were descriptive, and univariate and bivariate statistics were conducted in both phases. Items regarding the perceived impact of the COVID-19 pandemic on operations were collected using a 5-point Likert scale from 1 (“Strongly Agree”) to 5 (“Strongly Disagree”) (Appendix A). Items about concerns with meals service during COVID-19 were collected using a 4-point Likert scale from 1 (“No concern at all”) to 4 (“Serious concern”). Median scores and interquartile ranges (IQR) for the perceived impact of the COVID-19 pandemic on operations and concerns with meal service during COVID-19 were calculated among all sponsors. Subgroup analyses (e.g., by sponsor type) were not performed due to small sample sizes. Quantitative analyses were completed using the “dplyr” and “stats” packages in RStudio version 1.4.1106 and RStudio Desktop version 2022.07.2+576 [18,19].

## 3. Results

Sponsors reported a variety of factors that hindered or facilitated their operations in 2020 and 2021. The following sections describe:

1. Challenges and concerns due to the pandemic experienced by SFSP sponsors
2. Underlying reasons for the challenges and concerns experienced by SFSP sponsors during the pandemic
3. Importance of the USDA waivers

### 3.1. Challenges and Concerns Due to the Pandemic Experienced by SFSP Sponsors

In this section, we identify the challenges and concerns sponsors experienced during the pandemic, which are primarily informed by the quantitative results from the survey. Of the 50 SFSP sponsors in Maryland in 2020, 27 (54%) responded to the survey. Of the 51 SFSP sponsors in Maryland in 2021, 30 (59%) responded to the survey. Most survey respondents were public school sponsors (Phase I: 66.7%, Phase II: 63.3%) (Table 1). 

According to survey results in both phases, sponsors perceived the pandemic to have an impact on increasing their staff’s workload (Phase I: Median = 1.0, IQR = 1.5; Phase II: Median = 2.0, IQR = 2.0) (Table 2). In both phases, sponsors were moderately or seriously concerned about the safety of their staff (Phase I: Median = 4.0, IQR = 1.0; Phase II: Median = 4.0, IQR = 1.0) and students (Phase I: Median = 3.0, IQR = 1.0; Phase II: Median = 3.0, IQR = 2.0), and the potential that students will go hungry (Phase I: Median = 4.0, IQR = 1.0; Phase II: Median = 3.0, IQR = 2.0). 

Additionally, increased organizational spending due to the pandemic was perceived to be a challenge in both phases (Phase I: Median = 1.0, IQR = 1.5; Phase II: Median = 2.0, IQR = 2.0). Sponsors were moderately or seriously concerned about a dramatic decrease in meals served in 2020 but only a little concerned in 2021 (Phase I: Median = 2.0, IQR = 2.0; Phase II: Median = 3.0, IQR = 2.0). These worries aligned with sponsors’ reports in both phases that the pandemic caused an actual decrease in the number of meals served (Phase I: Median = 4.0, IQR = 2.5; Phase II: Median = 3.5, IQR = 1.0). In both years, sponsors were moderately concerned about the availability of product and/or distributor challenges (Phase I: Median = 3.0, IQR = 1.0; Phase II: Median = 3.0, IQR = 1.0). 

### 3.2. Underlying Reasons for the Challenges and Concerns Experienced by SFSP Sponsors during the Pandemic

The results in this section are informed by the qualitative data and explain reasons why sponsors experienced the challenges and concerns identified in the survey. In Phase II, most interview participants were public school sponsors (71%) (Table 3). In both years, approximately 55% of all SFSP sponsors in Maryland were public school sponsors. Of the 14 unique sponsors who participated in the interviews, five sponsors completed interviews in both phases.

#### 3.2.1. Staff Safety and Burnout

Qualitative findings from both phases revealed sponsors’ concern about staff availability/willingness to work was often tied to their perception that the pandemic increased staff workload, needing to hire more staff, and concerns about staff and student safety. Sponsors expressed that the pandemic affected their volunteer base on which they heavily rely. In 2020, one of the sponsors’ greatest concerns was the safety of their staff preparing/distributing meals; in 2021, this was the sponsors’ only serious concern. In 2020, sponsors had to adjust impromptu to the sudden school closures at the start of the pandemic, but by 2021, it appears sponsors had already adjusted. 

In both years, sponsors were still concerned about operating SFSP in ways that would keep staff—particularly their elderly staff—and students safe by maintaining physical distancing and other pandemic-related precautions. Staff availability and willingness to work were impacted by safety concerns, such as feeling uncomfortable and unsafe serving food due to concerns about contracting COVID-19. This impacted meal service as some sponsors had to close sites due to not having enough staff to run them. 

By 2021, sponsors felt that their staff were burnt out by operating SFSP continuously throughout the first year and a half of the pandemic. Sponsors reported needing to hire additional staff due to the pandemic and shared common concerns in the IDI, feeling worried that their “staff were exhausted by operating under the COVID exposures” (2021). With the transition to in-person service in 2021, sponsors described new challenges for their staff, such as delivering meals to classrooms. Some sponsors explained that many of their usual summer staff retired or did not return to work in 2021. One sponsor said, “Even the employees that normally work during the summer had no interest in working for various reasons. The district did offer an incentive for hourly employees that would work”. 

#### 3.2.2. Reasons for Changes in Participation

All sponsors changed their service models from in-person congregate feeding to grab-and-go, multiple-day food pickups, or a mix of these models during the initial pandemic response in 2020. Instead of hot meals, sponsors served cold meals and distributed boxes or bags with nonperishable food items, fresh produce, and frozen or pre-prepared meals. Sponsors described the importance of making the change to cold meals not only to maintain physical distancing safety measures during the pandemic but also to allow families to conveniently pick up multiple days’ worth of food in one trip. Many sponsors implemented hybrid models in 2021, serving a combination of hot meals and cold meals in congregate and non-congregate settings. Sponsors generally described a smooth transition to hybrid models because they had been serving meals during the pandemic under adapted models since March 2020. 

Although sponsors noticed increased participation at some sites, they also noticed decreased participation at other sites. In 2020, sponsors expressed concern about sites with low participation, knowing that the “need was still out there” (2020). Similar concerns persisted in 2021, as sponsors experienced decreased participation with the transition to in-person service and worried about children whose parents could no longer physically come to sites. Additionally, some sponsors felt that the “need” had changed due to contextual differences (e.g., adjusted to pandemic changes, policies and pandemic relief aimed at assisting families) in 2021 compared to 2020. 

Sponsors believed decreased participation was partially attributed to a lack of awareness of the availability and location of meal sites and difficulties with communication and outreach within their communities. In 2020, some sponsors especially felt that their reach was limited compared to others as their sites are not located where children live, and they operate independently of a school district. 

Qualitative findings in both phases revealed a connection between organizational spending and decreased participation (i.e., the number of meals served). Sponsors in both phases described difficulties with balancing their production of meals with unpredictable or fluctuating day-to-day participation or general decreased participation, which ultimately led to a financial deficit. Sponsors also noted dissatisfaction with the types of food served and lack of activities as key contributors to decreased participation. By virtue of serving with the grab-and-go model, sponsors were limited to serving a small selection of cold or frozen meals, particularly in 2020. Sponsors in both phases noticed “kids are just sick of the same thing” (2020) and “not enjoying this food anymore” (2021). 

#### 3.2.3. Importance of the Waivers

Sponsors highlighted the waivers as key facilitators that helped them overcome the aforementioned challenges and concerns. Sponsors expressed concerns about the uncertain extension of waivers beyond 2020 or 2021. In the IDI in both phases, sponsors were appreciative of the waivers, describing them as “extremely helpful” (2020) and “absolutely necessary” (2021). The waivers were extended into the 2020–2021 school year, and sponsors in 2021 continued to feel that the waivers were important and necessary to extend beyond 2021:

I want [policymakers] to consider the kids and the environments in which we are going into. To be mindful of these children, some of them really need this. If you were thinking about the kids, it will be free food for all children until the age of 18. It would not be, “You get lower reduced meals.” It would not be, “You have to be at the location.”

In both phases, sponsors emphasized the critical importance of the waivers to their operations and the ability to serve meals to more children. Even when sponsors reported decreased participation at their sites, they stated that their operations “would have been much worse without [the waivers]” (2021). Sponsors in both phases highlighted the following waivers as most important: area eligibility, mealtime flexibility, non-congregate feeding, and parent/guardian meal pickup. The area eligibility waiver allowed sponsors to serve meals in any area, regardless of how many households in the area had low incomes. The mealtime flexibility waiver allowed sponsors to serve meals outside of standard mealtimes. The non-congregate feeding waiver allowed sponsors to distribute meals in different models that did not require children to be gathered on-site (e.g., grab-and-go, pick-up). Finally, the parent/guardian meal pickup waiver allowed parents and guardians to pick up meals without their children present. One sponsor emphasized in 2021:

It would be nice to have [the waivers] all together. If we had to pick and choose, the top two for us are the kids did not have to eat on-site and that we can serve any child. If we could at least get those two, it would be a great thing. One without the other is like if you say you can serve kids anywhere, but then that means that you got to be worried [about], “Is this child on free or reduced?” We do not know. I would say we need all of them.

## 4. Discussion

This study aimed to use mixed methods to identify Maryland SFSP sponsors’ barriers and facilitators to operating the SFSP (quan) and explore the potential underlying explanations (QUAL) during the COVID-19 pandemic in 2020 and 2021. Although there is substantive empirical literature about the in-school nutrition programs (i.e., NSLP, SBP), there is little describing the out-of-school nutrition programs (i.e., SFSP). This study expands on the existing literature about the SFSP and out-of-school nutrition programs [6,20,21,22,23,24].

All sponsors were concerned about staff safety and increased workload due to the pandemic, and sponsors’ responses in the interviews elucidated the nuances of their concerns. For example, sponsors were specifically concerned about their volunteers who were vulnerable to contracting COVID-19. School nutrition professionals and food service directors in other studies reported similar concerns about staff safety during the pandemic [25,26,27]. Another example was sponsors’ awareness of staff burnout after working tirelessly and during a global pandemic, which led to challenges with staffing shortages and potentially closing sites. 

Concerns and challenges with changes in meal participation were often described by sponsors in our study. In 2020, the total number of meals served by Maryland SFSP sponsors significantly increased compared to summer 2019 despite decreases in the total number of sites and individual-site participation [6]. Yet, sponsors expressed concern about decreased meal participation during the pandemic for several reasons, such as lack of awareness which corroborates findings from an evaluation of a US school-based nutrition intervention program during the pandemic [28]. Sponsors in our study shared the belief that decreased contact with families and increased federal support impacted meal participation at their sites [28]. Another concern was the unpredictable fluctuations in meal participation which ultimately impacted organizational spending and potentially coming out with a deficit. This finding is aligned with the results from another US-based study of school food authorities reporting similar concerns with decreased participation and revenue [27].

At sites where participation decreased, sponsors were particularly worried about the students who were not coming out for a meal and potentially going hungry during the pandemic. For the last two years, free school meals have been offered to all students through USDA-issued waivers. However, aside from the permanent implementation of the non-congregate waiver in rural settings per the Consolidated Appropriations Act of 2023, there have been no announcements to date about extending flexibilities since they expired in June 2022. The results of our study and other similar studies demonstrate the importance of all SFSP waivers. Sponsors believe the waivers enabled them to feed children despite pandemic-related changes, and extending the waivers will be helpful [29,30]. Our findings also support advocacy efforts that call for Congress to extend the waivers and pass Healthy School Meals for All legislation [31,32].

### Strengths and Limitations

A strength of this study is its use of a sequential explanatory mixed methods design, where qualitative methods and data deepened our understanding of the barriers and facilitators to SFSP operation identified by sponsors in a quantitative survey. We aligned our mixed methodology with published guidelines for rigor [7,8,12,33]. Additionally, we collected and analyzed data over a consecutive two-year period, which enabled us to further explore complex patterns and themes that emerged from Phase I in Phase II. This provided a more in-depth analysis compared to cross-sectional studies.

This study has some limitations. The sample sizes used in both phases’ quantitative analyses were small, especially for our subgroups of sponsors. Public school sponsors’ experiences and perspectives may be overrepresented in the IDI. Although there was an overlap in the timing of the survey and the IDI in both phases, the sponsors’ time of survey completion and interview completion may not have aligned. It is possible that recall bias may have affected sponsors’ ability to accurately remember details about their experiences in the summer, particularly if they completed the survey and/or their interview in the fall or winter. Sponsors’ responses also may have been influenced by the timing of important announcements regarding SFSP waivers (i.e., waiver extensions) with their survey and/or interview completion. Given the possibility that our partners at MDHS might have pre-existing relationships with sponsors, they did not lead the interviews with study participants. However, it is possible that acquiescence bias affected sponsors’ responses in the surveys and interviews.

## 5. Conclusions

Understanding sponsors’ experiences during the pandemic may help inform policy changes to support SFSP operations in future out-of-school times, such as extending or making permanent waivers that bolster child nutrition programs. It is important to continue documenting and exploring the experiences of SFSP sponsors to inform future policy changes, particularly for emergency planning and anti-hunger efforts. 

## Figures and Tables

**Table 1:** Maryland 2020 and 2021 Summer Food Service Program (SFSP) sponsor survey respondent characteristics.

Sponsor Characteristics	Phase I: 2020 (*N* = 27)		Phase II: 2021 (*N* = 30)	
	n	%	n	%
Sponsor Type				
Public school food authority	18	66.7	19	63.3
Government agency	1	3.7	2	6.7
Nonprofit organization	7	25.9	6	20.0
Faith-based organization	1	3.7	2	6.7
Higher Education	0	0.0	1	3.3
Geography				
Urban	9	33.3	11	36.7
Rural	11	40.7	11	36.7
Both	7	25.9	8	26.7
Region of Maryland ^1^				
Central	7	25.9	11	36.7
Eastern Shore	6	22.2	5	16.7
Southern	9	33.3	5	16.7
Western	2	7.4	8	26.7
Multiple Regions	3	11.1	1	3.3
Number of years an SFSP sponsor (at time of survey completion) ^2^				
1–2 years	1	3.7	2	6.9
3–5 years	2	7.4	0	0
>5 years	24	88.9	27	93.1
Closed or Open sites				
Closed only	2	7.4	3	10.0
Open only	21	77.8	12	40.0
Both	4	14.8	15	50.0
Program Service Model ^3^				
In-person only	--	--	8	26.7
Distribution only	--	--	3	10.0
Hybrid (in-person and distribution)	--	--	19	63.3

^1^ Region based on the following map provided by the Maryland Department of Health: https://health.maryland.gov/dda/Pages/regional%20map.aspx (accessed on 11 October 2022). ^2^ In Phase II, *N* = 29 for this question because one sponsor did not provide a response. ^3^ This question was only included in the Phase II survey during summer 2021, when sponsors could serve in different ways. Phase I took place during summer 2020 when all sponsors served meals through the same model: distribution only.

**Table 2:** Sponsor responses to survey items regarding their perceived impact of and concerns due to the COVID-19 pandemic.

Construct from Survey ^1^	Phase I: 2020 (*N* = 27) Median (IQR)	Phase II: 2021 (*N* = 30) Median (IQR)
Perceived impact of the COVID-19 pandemic ^2^		
Increased staff workload	1.0 (1.5)	2.0 (2.0)
Increased organizational spending	1.0 (1.0)	2.0 (2.0)
Need to hire additional staff	3.0 (3.0)	2.0 (2.0)
Need to cut meals	3.0 (3.0)	3.5 (1.0)
Need to decrease the number of meals served at each site	4.0 (2.5)	3.5 (1.0)
Need to decrease the number of sites	3.0 (3.0)	3.0 (1.0)
Concerns due to the COVID-19 pandemic ^3^		
Safety of staff preparing/distributing meals	4.0 (1.0)	4.0 (1.0)
Safety of students/families when accessing meals	3.0 (1.0)	3.0 (2.0)
Staff availability/willingness to work	3.0 (1.0)	3.0 (1.0)
Transportation challenges for students & staff	3.0 (1.0)	3.0 (2.0)
Availability of product and/or distributor challenges	3.0 (1.0)	3.0 (1.0)
Regulatory restrictions on serving students during closures	3.0 (1.0)	3.0 (1.75)
Providing meals that do not have guaranteed reimbursement under SFSP	3.0 (1.0)	2.0 (2.0)
Potential that students will go hungry during summer	4.0 (1.0)	3.0 (2.0)
Dramatic increase in meals served	2.0 (2.0)	2.5 (1.0)
Dramatic decrease in meals served	4.0 (1.0)	2.0 (1.0)
Reduction in number of sites	3.0 (2.0)	2.0 (2.0)
Operating mobile meals	--	1.5 (2.0)

^1^ Interquartile range (IQR) reported as the difference between the first and third quartiles (Q3 − Q1). ^2^ Assessed on a 5-point Likert scale: 1 = Strongly Agree, 2 = Agree, 3 = Neither agree nor disagree, 4 = Disagree, 5 = Strongly Disagree. ^3^ Assessed on a 4-point Likert scale: 1 = No concern at all, 2 = Little concern, 3 = Moderate concern, 4 = Serious concern.

**Table 3:** Maryland 2020 and 2021 Summer Food Service Program (SFSP) sponsor interview participant characteristics.

Sponsor Characteristics	Phase I: 2020 (*N* = 12)		Phase II: 2021 (*N* = 7)	
	n	%	n	%
Sponsor Type				
Public school food authority	6	50	5	71
Government agency	1	8	0	0
Nonprofit organization	3	25	2	29
Faith-based organization	2	27	0	0
Geography				
Urban	2	27	0	0
Rural	6	50	2	29
Both (urban and rural)	4	33	5	71
Region of Maryland				
Central	3	25	1	14
Eastern Shore	3	25	1	14
Southern	3	25	2	29
Western	2	27	2	29
Multiple Regions	1	8	1	14
Number of years an SFSP sponsor ^1^				
>5 years	11	92	6	100
Closed or Open sites ^2^				
Closed only	1	8	0	0
Open only	8	67	5	71
Both	1	8	2	29
Program Service Model ^3^				
In-person only	--	--	0	0
Distribution only	--	--	0	0
Hybrid (in-person and distribution)	--	--	7	100

^1^ Region based on the following map provided by the Maryland Department of Health: https://health.maryland.gov/dda/Pages/regional%20map.aspx (accessed on 11 October 2022). ^2^ In Phase I, two sponsors did not serve meals during the pandemic, but we included them in our analysis because they had important insights regarding barriers to serving summer meals in summer 2020. ^3^ This question was only included in the Phase II survey during summer 2021, when sponsors could serve in different ways. Phase I took place during summer 2020 when all sponsors served meals through the same model: distribution only.

## Data Availability

Deidentified data may be available from the corresponding author upon request.

## Appendix A. Sponsor Survey Items

**Table A1:** 2020 and 2021 survey items regarding the perceived impact of the COVID-19 pandemic on operations.

Construct	Survey Item in 2020	Survey Item in 2021
Staff safety and burnout	The COVID-19 related operational changes to my program increased the workload for my staff.	The COVID-19 and summer 2021 related operational changes to my program will/have increase(d) the workload for my staff.
	I will/have need(ed) to hire additional staff this summer because of COVID-19 related operational changes to my program.	I will/have need(ed) to hire additional staff this summer because of COVID-19 and summer 2021 related operational changes to my program.
Reasons for changes in participation	The COVID-19 related operational changes to my program will/have increase(d) the amount of money my organization will need to spend to continue meals service in summer 2020	The COVID-19 and summer 2021 related operational changes to my program will/have increase(d) the amount of money my organization will need to spend to continue meal service in summer 2022.
	I will/have need(ed) to cut meals (either breakfast, lunch, supper, or snack) from sites due to COVID-19 related operational changes to my program.	I will/have need(ed) to cut meals (either breakfast, lunch, supper, or snack) from sites due to COVID-19 and summer 2021 related operational changes to my program.
	I will/have need(ed) to decrease the number of meals served at each site due to COVID-19 related operational changes to my program.	I will/have need(ed) to decrease the number of meals served at each site due to COVID-19 and summer 2021 related operational changes to my program.
	I will/have need(ed) to decrease the number of sites we serve due to COVID-19 related operational changes to my program.	I will/have need(ed) to decrease the number of sites we serve due to COVID-19 and summer 2021 related operational changes to my program.

**Table A2:** 2020 and 2021 survey items regarding concerns due to the COVID-19 pandemic.

Construct	Survey Item in 2020	Survey Item in 2021
Staff availability/willingness to work	Staff availability/willingness to work	Staff availability/willingness to work
	Safety of students/families when accessing meals	Safety of students/families when accessing meals
	Safety of staff preparing/distributing meals	Safety of staff preparing/distributing meals
	Transportation challenges for students & staff	Transportation challenges for students & staff
	Regulatory restrictions on serving students during closures	Regulatory restrictions on serving students during closures
Reasons for changes in participation	Dramatic increase in meals served	Dramatic increase in meals served
	Dramatic decrease in meals served	Dramatic decrease in meals served
	Reduction in number of sites	Reduction in number of sites
	Availability of product and/or distributor challenges	Availability of product and/or distributor challenges
	Providing meals that do not have guaranteed reimbursement under SFSP	Providing meals that do not have guaranteed reimbursement under SFSP
	Potential that students will go hungry during summer	Potential that students will go hungry during summer
		Operating mobile meal sites
